# Toxicokinetics and postmortem redistribution of amantadine in rats

**DOI:** 10.3389/fmed.2026.1783529

**Published:** 2026-02-27

**Authors:** Lingxiao Wang, Xiaomeng Sun, Chenxu Gao, Jinkai Wang, Jianbin Gong, Zhe Chen, Zhiwen Wei, Chao Zhang, Keming Yun

**Affiliations:** 1 School of Forensic Medicine, Shanxi Medical University, Taiyuan, China; 2 Technical Appraisal Research Center of the People's Procuratorate of Shanxi Province, Taiyuan, China; 3 Chengdu Public Security Bureau, Chengdu, China; 4 Xiyang County Public Security Bureau, Jinzhong, China; 5 Key Laboratory of Forensic Toxicology, Ministry of Public Security, Beijing, China

**Keywords:** amantadine, dynamic distribution, forensic toxicology, male rats, postmortem redistribution

## Abstract

**Objectives:**

Amantadine (AMD) is an antiviral and antiparkinsonian drug with a narrow therapeutic window and a recognized risk of severe intoxication. Interpretation of postmortem drug concentrations is complicated by postmortem redistribution (PMR), yet systematic toxicokinetic and multi-tissue PMR data for amantadine remain limited.

**Methods:**

An integrated investigation of amantadine toxicokinetics and postmortem redistribution was conducted in male rats. For toxicokinetic assessment, a single oral dose of 450 mg/kg (LD50) was administered, and concentrations were quantified in blood and nine tissues over a 96-h period. For the PMR study, rats received low (42 mg/kg), medium (LD50), and high (2 × LD50) doses, followed by controlled postmortem storage at 4 °C and 20 °C for up to 96 h. Amantadine concentrations were determined using validated HPLC–MS/MS methods and analyzed by pharmacokinetic and statistical approaches.

**Results:**

Amantadine was rapidly absorbed and widely distributed, exhibiting pronounced tissue-specific heterogeneity. The liver and kidney showed the highest exposure, whereas accumulation in the brain and testis was limited. Postmortem redistribution was substantial and tissue dependent, and was strongly influenced by dose, postmortem interval, and storage temperature. Blood concentrations were unstable over time, while solid organs, particularly the liver and spleen, exhibited higher and more sustained postmortem concentrations. Notably, selected inter-tissue concentration ratios (e.g., liver-to-lower limb muscle and spleen-to-brain) displayed consistent, dose-dependent trends across postmortem conditions.

**Conclusions:**

This study provides a comprehensive characterization of amantadine toxicokinetics and postmortem redistribution across multiple biological matrices. The findings underscore the limitations of relying solely on postmortem blood concentrations and support the complementary use of selected tissues and inter-tissue concentration ratios as comparative indicators in toxicological interpretation. These results offer mechanistic insight into postmortem drug dynamics and provide practical reference data to improve the interpretation of suspected amantadine intoxication.

## 1 Introduction

Amantadine (AMD) is a well-established antiviral and antiparkinsonian agent with a long history of clinical use, resulting in sustained human exposure and a persistent risk of misuse and overdose (1–7). In recent years, renewed interest in AMD has emerged due to its reported antiviral activity against emerging viruses and its documented misuse in animal husbandry, particularly in poultry farming (8–10). Together, these factors increase the likelihood of both therapeutic and non-therapeutic exposure, highlighting the toxicological and public health relevance of this compound.

From a pharmacokinetic perspective, AMD is predominantly eliminated unchanged via renal excretion, with minimal metabolic transformation (11). Its therapeutic dosing range lies close to the threshold of toxicity, and overdose—particularly in combination with anticholinergic agents—can lead to severe central nervous system toxicity, including hallucinations, delirium, coma, and death (12, 13). While the clinical pharmacokinetics of AMD at therapeutic doses have been described, systematic data addressing its toxicokinetics following overdose remain limited, especially regarding its tissue distribution—that is, its specific partitioning into different organs (14). Several central questions remain to be elucidated, including systematic, time-resolved organs distribution data at toxic exposure levels, dose-dependent characterization of postmortem redistribution (PMR) in tissues, and controlled evaluation of temperature- and interval-dependent PMR.

In fatal intoxication, postmortem drug concentrations may not accurately reflect antemortem levels due to PMR, a process characterized by time-, tissue-, and condition-dependent concentration changes after death. PMR is influenced by multiple factors, including physicochemical properties, tissue affinity, apparent volume of distribution, and postmortem processes such as cellular autolysis and diffusion (15). Empirical indicators, such as cardiac-to-peripheral blood or liver-to-blood concentration ratios, are commonly applied to evaluate the potential impact of PMR; however, their applicability is highly compound specific and remains insufficiently validated for many drugs (16–19).

Amantadine exhibits several properties associated with a high propensity for PMR, including a large apparent volume of distribution and weakly basic characteristics that favor tissue sequestration. In addition, practical factors such as delayed body discovery, transportation, and sampling may allow postmortem redistribution to progress before specimen collection, further complicating interpretation. Consequently, a detailed understanding of both the *in vivo* tissue distribution of AMD at toxic doses and its postmortem redistribution behavior under defined conditions is essential for improving the reliability of toxicological interpretation.

Accordingly, the present study systematically investigated the toxicokinetics and tissue distribution of AMD in a rat model following intragastric administration at a toxic dose, as well as its postmortem redistribution in blood and multiple organs across defined postmortem intervals and storage temperatures. By integrating toxicokinetic analysis with controlled PMR modeling, this study aims to provide mechanistic insight into postmortem drug dynamics and to generate comparative reference data to support the interpretation of severe and fatal amantadine intoxication.

## 2 Materials and methods

### 2.1 Chemicals and reagents

Adamantan-1-amine and Amantadine-D6 were obtained from Shanghai Macklin Biochemical Technology Co., Ltd (Shanghai, China). HPLC grade formic acid was purchased from Tianjin Damao Chemical Reagent Partnership Enterprise (Tianjin, China). HPLC grade acetonitrile and methanol were purchased from Merck (Darmstadt, Germany). Molecular formula, CAS number, SMILES ID and InChi code of Adamantan-1-amine and Amantadine-D6 each are listed in Supplementary Table 1.

### 2.2 Animals

Male Sprague-Dawley rats (6 weeks old, weighing 180–220 g), were obtained from the SPF (Beijing) Biotechnology Co., Ltd. (Beijing, China). The animals were group-housed (five per cage) under standard laboratory conditions with controlled temperature and humidity, and a 12 h light/dark cycle (lights on 07:00–19:00). Food and drinking water were provided *ad libitum*. All animal experiments complied with the ARRIVE guidelines, were approved by Institutional Animal Care and Use Committee of Shanxi Medical University [approval Nos. 2021GLL051 (March 3, 2021)], and performed in accordance with the current relevant legislation in China.

### 2.3 Study design

#### 2.3.1 The dynamic distribution study of AMD in male rats

Prior to the experiment, all rats were acclimated in the animal housing room for 1 week. Sixty rats were randomly assigned into 10 groups (*n* = 6). A toxic dose of AMD (450 mg/kg, equivalent to 1/2 LD50) was administered to all rats by intragastric gavage (i.g.). Rats from each group were euthanized by cervical dislocation at designated time points: 0.5, 1, 2, 4, 8, 12, 24, 48, 72, and 96 h post-administration. Immediately after euthanasia, samples of heart blood, peripheral blood, heart, liver, spleen, lung, kidney, brain, lower limb muscle, and testis were collected for subsequent AMD quantification.

#### 2.3.2 The PMR study of AMD in rats

A total of 252 rats were administered AMD via i.e., at three dose levels low (42 mg/kg), medium (LD50), and high (2 × LD50) to investigate its postmortem redistribution in tissues. To standardize postmortem intervals (PMI), animals surviving beyond the mean time of death in the medium-dose group were euthanized at that time point. Within each dose group, rats were subdivided into seven PMI subgroups (*n* = 12 per subgroup), with each subgroup equally allocated to two storage conditions: 4 °C or 20 °C (*n* = 6 per condition) in constant-temperature incubators.

### 2.4 Sample preparation

A 50 μL aliquot of blood or 50 mg of thoroughly homogenized tissue was weighed or measured into a 1.5 ml centrifuge tube. Subsequently, 5 μL of the internal standard (amantadine-D6, 100 μg/ml) and 950 μL of methanol were added to each tube. The mixture was vortexed for 30 s, then centrifuged at 15,294 × g for 10 min at 4 °C. Following centrifugation, a 400 μL aliquot of the supernatant was transferred and filtered through a 0.22 μm organic phase filter membrane. Finally, 3 μL of the filtrate was injected into the high-performance liquid chromatography-tandem mass spectrometry (HPLC-MS/MS) system for analysis. Samples exceeding the quantification range were diluted before injection.

### 2.5 Apparatus

HPLC-MS/MS conditions, including chromatographic, instrumentation, and mass spectrometric parameters, are detailed in Supplementary Tables 2, 3.

### 2.6 Method validation

The selectivity, calibration curve, sensitivity, accuracy, precision, recovery, stability, dilution effects were evaluated according to FDA guidelines (20). Detailed procedures and calibrators for the standard addition approach are provided in the Supplementary material.

### 2.7 Data statistical analysis

All data are presented as the mean ± standard error of the mean (SEM), unless otherwise stated. Statistical analyses were performed using SPSS (version 26.0; IBM Corp., Armonk, NY, United States) and GraphPad Prism (version 8.0.2; GraphPad Software, San Diego, CA, United States). Pharmacokinetic parameters were calculated using Phoenix WinNonlin (version 8.1; Certara United States, Inc., Princeton, NJ, United States).

A hierarchical analytical strategy was adopted. For dynamic tissue distribution, a univariate general linear model (GLM) evaluated the main effects of time and tissue type, and their interaction, on AMD concentration. Homogeneity of variances was assessed using Levene's test. Significant effects (*p* < 0.05) were further analyzed as follows: (1) Temporal changes within each tissue were assessed using Welch's ANOVA followed by Games-Howell *post-hoc* tests. (2) Differences between tissues at each time point were analyzed with one-way repeated-measures ANOVA, applying Greenhouse-Geisser correction when Mauchly's test indicated violation of sphericity. Bonferroni-corrected paired *t*-tests were used for pairwise comparisons.

Postmortem redistribution data were analyzed using a linear mixed-effects model, with dose, storage temperature, PMI, tissue type, and all two-way interactions as fixed effects, and a random intercept for individual rats. For significant interactions, *post-hoc* comparisons of estimated marginal means were performed with Bonferroni correction (α = 0.05). Temporal dynamics under each combination of dose, temperature, and tissue were further evaluated with Welch's one-way ANOVA, followed by Games-Howell pairwise tests.

Pharmacokinetic parameters derived from the concentration-time data included elimination half-life (*t*1/_2_), area under the concentration-time curve from time zero to the last measurable time point (AUC_0−*t*_), apparent clearance (CL/F), volume of distribution (Vz), time to reach maximum concentration (*T*_max_), maximum concentration (*C*_max_), and mean residence time up to the last measurable point (MRT_last).

## 3 Results

### 3.1 Method validation

The developed HPLC-MS/MS method for AMD quantification exhibited robust specificity, with no endogenous interference observed in the biological matrices. Calibration curves demonstrated excellent linearity over the ranges of 1–1,000 ng/ml in blood (*R*^2^ > 0.995) and 0.1–100 ng/mg in tissue (*R*^2^ > 0.995).

Precision and accuracy were within acceptable limits. Intra- and inter-day precision, expressed as relative standard deviation (RSD), ranged from 6.80 to 13.78%, while accuracy, expressed as relative error (RE), ranged from 78.63 to 112.67%. Dilution integrity was confirmed, with measured concentrations within 91.27–112.44% for blood and 87.45%−107.62% for tissue of expected values.

Mean extraction recoveries for AMD from spiked samples were 79.4–94.4% (RSD ≤ 7.3%) in blood and 82.4–88.2% (RSD ≤ 6.9%) in tissues. Matrix effects were moderate, ranging from 77.3 to 87.1% (RSD ≤ 5.1%) in blood and 79.6 to 87.1% (RSD ≤ 6.1%) in tissues. Stability assessments, including freeze-thaw cycles and autosampler storage, showed deviations below 15%, consistent with standard bioanalytical guidelines.

Complete validation data are summarized in Supplementary Table 4.

### 3.2 The dynamic distribution of AMD in male rats

Following AMD administration via i.e., at a dose of 1/2 LD50, distinct concentration–time profiles were observed across cardiac blood, heart, liver, spleen, lungs, kidneys, lower limb muscle, brain, and testicular tissues (Supplementary Table 5). A two-way general linear model (GLM) revealed significant main effects of time [*F*_(9, 450)_ = 106.579, *p* < 0.001] and tissue type [*F*_(8, 450)_ = 207.395, *p* < 0.001] on AMD concentrations, with a significant time × tissue interaction [*F*_(72, 450)_ = 6.686, *p* < 0.001], indicating that temporal concentration patterns were tissue-dependent. Accordingly, further analyses were conducted to assess temporal changes within each tissue and to compare concentrations between tissues at each time point.

**Figure 1:**
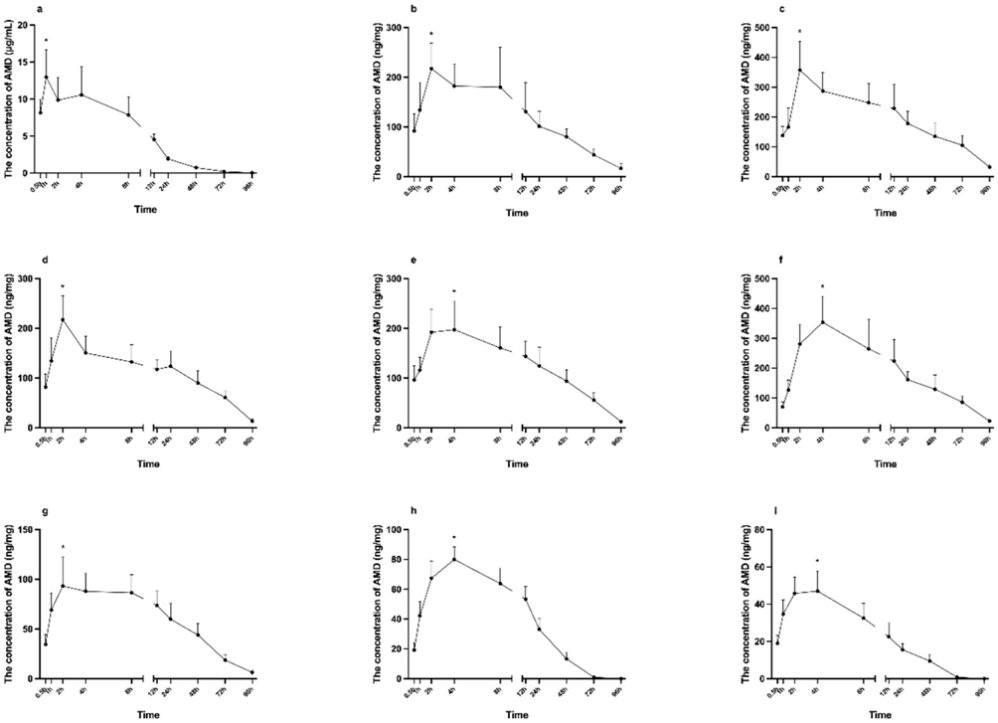
The dynamic distribution of AMD in rats following a dose of 1/2 LD_50_. Cardiac blood and Tissues were collected at the indicated time points. Concentrations of AMD in cardiac blood **(a)**, heart **(b)**, liver **(c)**, spleen **(d)**, lung **(e)**, kidney **(f)**, brain **(g)**, lower limb muscle **(h)** and testis **(i)** are shown. Data was represented by mean ± SEM. *p < 0.05 compared to both adjacent time points in AMD concentration.

In cardiac blood, AMD was rapidly absorbed, reaching a peak concentration of 12.97 ± 3.6 μg/ml at 1 h post-administration (significantly higher than 0.5 h and 2 h, *p* < 0.05), followed by a general decline, with a minor transient increase at 4 h. AMD remained detectable throughout the 96-h study period, with a final concentration of 39.55 ± 22.02 ng/ml (Figure 1a).

All other tissues showed significant time-dependent changes (*p* < 0.05). Peak concentrations in the heart, liver, spleen, and brain occurred at 2 h (217.34 ± 51.3, 357.75 ± 95.8, 217.45 ± 47.7, and 93.0 ± 29.5 ng/mg, respectively; Figures 1b–d, g), followed by rapid decline. In contrast, lung, kidney, lower limb muscle, and testis peaked at 4 h (197.3 ± 57.2, 353.48 ± 87.0, 79.95 ± 8.55, and 47.02 ± 10.8 ng/mg, respectively; Figures 1e, f, h, i), then gradually decreased.

The peak concentrations of AMD across various tissues were ranked as follows: cardiac blood > liver > kidney > spleen > heart > lung > brain > lower limb muscle > testis. Concentrations in lower limb muscle and testis fell near the lower limit of quantification by 72 h and became undetectable by 96 h.

### 3.3 The PMR of AMD in male rats

**Figure 2:**
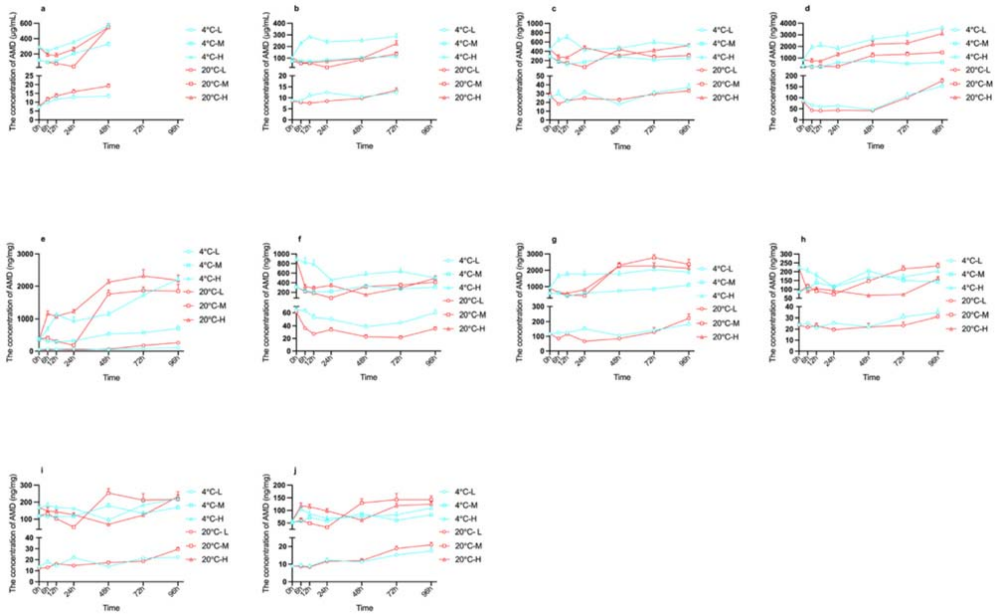
The PMR of AMD in rats following three dose levels low (42 mg/kg), medium (LD50), and high (2 × LD50). Figures show the PMR over time and temperature in peripheral blood **(a)**, cardiac blood **(b)**, heart **(c)**, liver **(d)**, spleen **(e)**, lung **(f)**, kidney **(g)**, brain **(h)**, lower limb muscle **(i)**, and testis **(j)**.

Following fatal intoxication induced by intragastric administration of AMD, distinct postmortem concentration trends were observed across tissues. Linear mixed-effects model analysis identified significant main effects of postmortem interval [*F*_(6, 221.384)_ = 22.323, *p* < 0.001], tissue type [*F*_(9, 2005.053)_ = 137.208, *p* < 0.001], and dose [*F*_(2, 219.188)_ = 323.564, *p* < 0.001] on AMD concentration. In contrast, the main effect of storage temperature was not significant [*F*_(1, 218.935)_ = 0.125, *p* = 0.724]. Significant two-way interactions (time × tissue, dose × tissue, time × dose, time × temperature, dose × temperature, temperature × tissue) highlighted the multifactorial complexity of AMD redistribution. The corresponding concentration changes are detailed in Figure 2.

Peripheral blood concentrations were dose- and temperature-dependent. At 4 °C, all dose groups showed gradual increases over 48 h, with 48h/0h ratios of 1.71–2.81 (Supplementary Table 6). At 20 °C, the M group displayed an initial decrease by 24 h followed by a rebound at 48 h, whereas L and H groups followed increasing trends. Cardiac blood exhibited similar dose-dependent PMR patterns, with the H group showing the largest magnitude. Notably, the M group at 20 °C initially decreased before recovering, paralleling the peripheral blood pattern.

PMR in the heart was limited, mainly dose-dependent, with minimal temperature influence. The H group at 4 °C peaked within the first 12 h postmortem before returning to baseline. Lung concentrations remained relatively stable in L and M groups at 4 °C, while H groups showed modest decreases by 24 h. At 20 °C, lung PMR was accelerated, with the M group reaching a minimum at 24 h before a partial recovery.

The liver exhibited higher concentrations and larger postmortem increases of AMD compared with other tissues. (Figure 2d). In the L group, AMD concentrations decreased significantly from 0 to 48 h (*p* < 0.05) and subsequently increased between 72 and 96 h (*p* < 0.05), with no obvious temperature dependence. In contrast, in the M and H groups, the postmortem increase occurred more slowly at 20 °C than at 4 °C. Specifically, a significant increase was observed at 48 h under 20 °C (0 h vs. 48 h, *p* < 0.05), compared with 24 h under 4 °C (0 h vs. 24 h, *p* < 0.05), highlighting the combined influence of dose and storage temperature on hepatic PMR.

The spleen exhibited the most pronounced PMR among all tissues examined (Figure 2e). At 4 °C, concentration ratios relative to 0 h increased progressively over time across all dose groups. In the H group, the ratio rose from 1.92 at 6 h to 3.72 at 12 h, reaching a peak of 6.06 by 72 h. The M and L groups showed similar progressive increases, attaining ratios of 4.81 at 72 h. At 20 °C, redistribution was markedly accelerated and intensified. In the H group, the ratio increased from 3.19 at 6 h to 5.12 at 72 h, while the M group exhibited a rapid rise from 4.89 at 48 h to 6.44 at 72 h, one of the highest values recorded in this study. Notably, even the L group reached 4.89 by 48 h, illustrating that temperature markedly accelerates and amplifies splenic PMR across all doses.

Kidney PMR resembled that of the lung, with rapid concentration increases in H group at 4 °C and dose-dependent, temperature-sensitive patterns at 20 °C. Brain concentrations remained stable in L group. M and H group displayed biphasic or delayed increases, with temperature influencing the timing and magnitude of peaks (e.g., 20 °C accelerated early decline and rebound in H group).

The postmortem redistribution (PMR) patterns of AMD were comparable between lower limb muscle and testicular tissues (Figures 2i,j). In the L group, concentrations increased gradually over the 96-h period. The M group exhibited a significant increase by 48 h (24 h vs. 48 h, *p* < 0.05). In contrast, the H group demonstrated a biphasic response: a decrease at 48 h followed by a subsequent increase (24 h vs. 48 h and 48 h vs. 72 h, *p* < 0.05).

## 4 Discussion

### 4.1 The dynamic distribution of AMD in male rats

Following a single i.e., dose (450 mg/kg) of AMD in rats, marked inter-organ heterogeneity in pharmacokinetic parameters was observed, reflecting tissue-specific distribution, retention, and elimination mechanisms. The data presented in Table 1 indicate that AMD exhibits rapid absorption, extensive tissue penetration, and prolonged retention in specific organs-particularly the liver and kidney-while showing restricted exposure in the brain and testes despite measurable penetration across protective barriers.

**Table 1:** Pharmacokinetic parameters of AMD in tissues of male rats.

**Pharmacokinetic parameters**	**Cardiac blood**	**Heart**	**Liver**	**Lung**	**Spleen**	**Kidney**	**Brain**	**Lower limb muscle**	**Testis**
t_1/2_ (h)	12.07	21.06	34.40	16.45	31.73	28.61	17.21	11.32	13.54
AUC_0 − t_ (μg/mL^*^h or ng/g^*^h)	185.45	7,802.98	1,3803.72	8,787.93	8,394.94	1,2790.96	4,062.73	1,992.10	1,069.00
CL/F (mL/h/kg)	2,417.48	54.16	29.18	49.54	49.98	32.81	106.62	224.05	414.55
Vz (L/kg)	42.08	1.65	1.45	1.18	2.29	1.35	2.65	3.66	8.10
C_max_ (μg/mL or ng/mg)	12.97	217.34	357.75	197.32	217.45	353.48	92.99	79.95	47.02
MRT_last (h)	16.18	38.14	46.92	36.33	42.43	40.45	34.65	20.08	21.72
T_max_ (h)	1	2	2	4	2	4	2	4	4

t_1/2_, elimination half-life; AUC_0 − t_, area under the concentration-time curve; CL/F, clearance; Vz, distribution volume; *C*_max_, maximum concentration; MRT_last, mean residence time; *T*_max_, time of maximum concentration.

AMD was rapidly absorbed from the gastrointestinal tract, achieving peak plasma concentrations (*C*_max_ = 12.97 μg/mL) within 1 h post-dose (*T*_max_ = 1 h), consistent with its known high oral bioavailability and lipophilic nature (21). This rapid absorption profile is corroborated by previous rodent studies demonstrating near-complete absorption within 1–2 h after oral administration (22, 23). The high systemic exposure (AUC0–t = 185.45 μg·h/ml in cardiac blood) further supports efficient transmucosal diffusion through the gastric and intestinal epithelium, facilitated by AMD's amphipathic structure and minimal first-pass metabolism (24, 25). Consistent with the findings reported by Burnat et al., AMD readily crosses the blood–brain barrier, with peak blood concentrations achieved within 1 h after administration. At this time point, brain drug concentrations were approximately fourfold higher than those in blood (26).

The liver and kidneys exhibited the highest total drug exposure (AUC_0−−*t*_ > 1.2 × 104 ng·h·mg^−1^) and prolonged terminal half-lives (t1/_2_≈ 28–34 h). These findings are consistent with previous reports demonstrating rapid accumulation of radiolabeled AMD in hepatic and renal tissues within hours after administration (27). The combination of high tissue perfusion, carrier-mediated uptake, and lysosomal sequestration likely accounts for the large AUC and prolonged mean residence times (MRTs) observed in this study. The hepatic-to-blood AUC ratio in our study (>70-fold) exceeds that reported by Oliveira et al. (7), which may reflect the substantially higher oral dose administered (450 mg·kg^−1^ vs. 50 mg·kg^−1^), as well as species- or formulation-dependent differences in absorption.

The overall t_1_/__2__ and MRT_last ranged widely—from 11 h and 20 h in lower limb muscle to nearly 35 h and 47 h in liver—indicating distinct compartmental retention and clearance mechanisms. Collectively, these results reflect the physicochemical properties of AMD, a lipophilic weak base (pKa ≈ 10.8) with moderate membrane permeability and predominantly renal excretion (28, 29).

Goralski and deVries et al. have demonstrated that AMD is primarily eliminated through active renal tubular secretion and reabsorption mediated by organic cation transporter 2 (OCT2) and multidrug and toxin extrusion proteins (MATE1/2-K) (11, 30). You et al. performed an animal study to evaluate the target tissues for monitoring AMD abuse in broiler chickens and reported that the highest residue concentration were observed in the liver, with slow elimination and detectable residues persisting until the end of the experiment. In contrast, drug residues in breast lower limb muscle and plasma were eliminated more rapidly. These findings are consistent with the present results, despite differences in the species studied (31).

### 4.2 The PMR of AMD in male rats

The present findings demonstrate that AMD undergoes marked, tissue-specific postmortem redistribution in rats, even under controlled storage temperatures (32). The significant effects of time, tissue type, and dose, together with strong interaction terms, confirm that PMR is governed by dynamic, matrix-dependent processes rather than simple passive diffusion (33). The linear mixed-effects model analysis revealed a complex interplay among the factors influencing AMD concentration, as evidenced by several significant two-way interactions (all *p* < 0.001).

Most notably, the significant interaction between postmortem interval and tissue type indicates that the postmortem kinetic trajectory of AMD is strongly tissue specific, with individual organs exhibiting distinct temporal redistribution patterns. This finding underscores that AMD does not follow a uniform postmortem fate but instead responds to organ-dependent physiological and structural characteristics. This tissue specificity is further reinforced by the robust interaction between dose and tissue type, demonstrating that tissue capacity to accumulate and retain AMD varies nonlinearly with the administered dose, consistent with tissue-specific saturation phenomena and differential intracellular binding or sequestration capacities.

In addition to intrinsic tissue properties, postmortem AMD kinetics were modulated by external conditions. Interactions involving time, dose, and temperature indicate that both the rate and direction of redistribution are jointly influenced by the initial body burden and the postmortem environment, with elevated storage temperatures accelerating concentration changes and higher doses amplifying redistribution magnitude. Importantly, the interaction between temperature and tissue type highlights that thermal sensitivity is not uniform across biological matrices. Collectively, these interactions demonstrate that postmortem AMD concentrations arise from the combined and interdependent effects of tissue characteristics, dose level, postmortem interval, and environmental temperature, rather than being governed by any single dominant factor.

Blood specimens exhibited particularly pronounced instability. Both peripheral and cardiac blood showed progressive concentration increases under refrigerated conditions. In the L group stored at 4 °C, peripheral blood AMD concentrations increased from 7.84 μg/ml at 0 h to 13.43 μg/ml at 48 h, while cardiac blood rose from 8.64 μg/ml to 12.44 μg/ml over a similar interval, indicating substantial postmortem influx of drug from surrounding tissues (34). These findings are consistent with the classical concept that blood acts as a secondary compartment that equilibrates with drug-rich organs after death (35). Although cardiac blood is traditionally considered more vulnerable to PMR due to its proximity to solid organs, the present data demonstrate that peripheral blood may be similarly affected, particularly for basic amines such as AMD (36).

At 20 °C, a biphasic redistribution pattern became evident, especially in the M group. Peripheral blood concentrations declined sharply from 116.47 μg/ml at 0 h to 36.32 μg/ml at 24 h, followed by a dramatic rebound to 547.42 μg/ml at 48 h. Such early postmortem decreases followed by delayed increases have been described for other weakly basic, lysosomotropic xenobiotics. This kinetic pattern may be attributable to an initial phase of sequestration via cellular uptake and clot formation, with subsequent secondary release during autolysis, though this specific process was not directly tested (37). This phenomenon underscores the highly dynamic nature of PMR and the difficulty of interpreting single postmortem blood measurements (38).

In contrast to blood, the liver appeared to function as a major site of AMD retention, exhibiting characteristics consistent with a reservoir tissue. In the L group at 4 °C, hepatic AMD concentrations decreased from 76.02 ng/mg at 0 h to 47.17 ng/mg at 48 h, before increasing to 152.68 ng/mg at 96 h. This delayed secondary rise is consistent with the known propensity of weak bases to accumulate within acidic intracellular compartments such as lysosomes, followed by gradual release as membrane integrity deteriorates postmortem (39). Highly perfused parenchymal organs with large intracellular volumes, including the liver, are recognized as principal contributors to PMR (40).

Among all solid organs examined, the spleen showed the most extreme redistribution behavior. At 20 °C in the L group, splenic concentrations increased from 51.69 ng/mg to 264.63 ng/mg over 96 h, representing more than a five-fold increase. We hypothesize that the pronounced PMR may be attributed to the spleen's high blood content and its close anatomical proximity to the stomach in rats. Following intragastric dosing, the stomach may act as a substantial postmortem drug reservoir due to residual gastric contents. Progressive autolysis and hemolysis may further facilitate the diffusion of AMD from gastric and splanchnic compartments into adjacent highly perfused parenchymal organs, such as the spleen, thereby potentially amplifying the magnitude of splenic PMR observed in this model (19, 41).

Although classic PMR theory identifies the myocardium as a major postmortem drug reservoir for many cardioactive and highly lipophilic compounds, including digoxin, tricyclic antidepressants, and calcium channel blockers, this framework is not universally applicable to all xenobiotics (39, 42). In the present study, cardiac tissue did not function as a stable long-term reservoir for AMD; instead, it exhibited limited, heterogeneous, and temporally dynamic postmortem concentration fluctuations. Notably, the changes in heart tissue concentration remained within a relatively narrow range and were comparatively more stable than those detected in other tissues. Pulmonary tissues displayed limited but temperature-dependent postmortem redistribution, with an early decline in concentrations followed by a partial rebound at elevated temperatures, consistent with temperature-driven tissue degradation and predominantly unidirectional drug efflux (43, 44).

The PMR of AMD in kidney displayed a clear dose- and temperature-dependent profile. At 4 °C, concentrations in the L and M groups remained largely stable, consistent with suppression of enzymatic autolysis and delayed cellular degradation under refrigerated conditions, which limits postmortem solute mobility (39, 45). In contrast, the rapid approximately two fold increase observed in the H group within 6 h suggests that steep concentration gradients can drive early redistribution even when overall tissue breakdown is minimal (42). At 20 °C, accelerated autolysis and membrane disruption increased intrarenal solute mobility, resulting in an initial concentration decline followed by delayed back-diffusion as tissue integrity deteriorates. This mechanism explains the biphasic pattern at low dose and the marked increases at 48 h in H groups (32).

Brain concentrations were comparatively stable, particularly in the L group, supporting the suitability of postmortem brain as a relatively robust matrix for xenobiotic quantification (46). However, the structural integrity of brain cells and their membranes progressively deteriorate during the postmortem interval, a process accelerated by elevated temperature, which alters tissue microstructure and solute mobility (47). Consequently, temperature strongly influences postmortem analyte behavior in the brain; lower temperatures preserve AMD by slowing enzymatic and autolytic processes, whereas higher temperatures accelerate degradation and redistribution (48).

Lower limb muscle and testis have been proposed as alternative specimens in postmortem toxicology because they are abundant and undergo decomposition later than blood or visceral organs. Moreover, sampling from peripheral regions minimizes the influence of diffusion from drug-rich organs such as the stomach, liver, and lungs. (49). Although generally considered more stable and less prone to PMR (50), these tissues still demonstrate measurable PMR under conditions of a high drug dose and a prolonged postmortem interval. This effect is likely driven by passive diffusion from residual blood, facilitated by initial postmortem capillary perfusion and the high-water content of these tissues, which promotes penetration of hydrophilic weak bases. Minor autolysis and postmortem pH shifts may further modulate drug partitioning, enabling transient accumulation even in peripheral tissues.

In this controlled study, the L group represented upper-range therapeutic exposure to AMD, whereas the H group corresponded to a severe intoxication level (2 × LD50). Several inter-tissue concentration ratios showed consistent dose-dependent divergence. At 4 °C, the heart-to-testis ratio was < 3 in the L group but exceeded three in the high-dose group, whereas the liver-to-testis ratio remained < 10 at low dose and exceeded 10 at high dose. At 20 °C, similar shifts were observed: the heart-to-brain ratio remained < 2 at low dose but exceeded two at high dose, and both the spleen-to-brain and liver-to-lower limb muscle ratios were < 10 at low dose but exceeded 10 at high dose. In contrast, the brain-to-lower limb muscle ratio exceeded one at low dose but declined below one at high dose.

These systematic reversals suggest that solid organs—such as the spleen, liver, and heart, which are major AMD accumulation sites and anatomically closer to the stomach—undergo limited PMR at therapeutic exposures but pronounced redistribution at high tissue burdens. Conversely, the brain, a pharmacological target organ located farther from the stomach, displayed relatively greater redistribution at lower doses and more constrained changes under extreme exposure. Collectively, these dose-dependent inter-tissue ratios may provide supportive experimental evidence for distinguishing therapeutic from severe or lethal AMD intake in forensic toxicological interpretation. However, the inter-tissue ratios observed in our controlled model provide preliminary clues. However, whether they can reliably help infer the premortem dose in real-case scenarios needs further investigation. A key limitation of this study is the use of a male-only rat model, which precludes the assessment of potential sex-based differences in AMD handling. Given the well-documented sex differences in pharmacokinetics, the applicability of our findings to females remains unknown and warrants future investigation.

## 5 Conclusion

We characterized the dynamic tissue distribution and postmortem redistribution (PMR) of AMD in male rats by integrating *in vivo* pharmacokinetics with controlled postmortem models across a range of doses, temperatures, and postmortem intervals. Our results demonstrate pronounced tissue-specific retention *in vivo* and complex, condition-dependent PMR after death, driven primarily by dose-dependent saturation kinetics, tissue physiology, and ambient temperature. A key finding is the identification of reproducible, dose-dependent divergence patterns in critical inter-tissue concentration ratios (e.g., heart-to-brain, liver-to-lower limb muscle), which may serve as a promising potential indicator for distinguishing between therapeutic and fatal AMD exposures in forensic contexts. Furthermore, the relative stability of brain concentrations at lower doses and the pronounced accumulation capacity of the liver and spleen offer valuable guidance for matrix selection in postmortem analysis. While limitations include the use of a single species under controlled experimental conditions, this study provides essential mechanistic insights and actionable reference data. Collectively, these findings provide a basis for more reliable interpretation of postmortem AMD concentrations and identify promising tissue-ratio markers for future validation in forensic casework.

## Data Availability

The original contributions presented in the study are included in the article/Supplementary material, further inquiries can be directed to the corresponding author.
