# Supplementary material for: Toxicokinetics and postmortem redistribution of amantadine in rats
Source: Front Med (Lausanne). 2026 Feb 27;13:1783529. doi: 10.3389/fmed.2026.1783529 (PMC12982071; doi:10.3389/fmed.2026.1783529)
Supplement: Supplementary file 1 [file Table_1.docx]

Materials and Methods

**Supplementary Table 1:** Molecular formula, CAS number, SMILES ID and InChi code of Adamantan-1-amine and Amantadine-d6 each.

|  | Molecular formula | CAS number | SMILES ID | InChi code |
| --- | --- | --- | --- | --- |
| Adamantan-1-amine (Amantadine) | C10H17N | 768-94-5 | C1C2CC3CC1CC(C2)(C3)N | InChI=1S/C10H17N/c11-10-4-7-1-8(5-10)3-9(2-7)6-10/h7-9H,1-6,11H2 |
| Amantadine-d6 | C10H11D6N | 1219805-53-4 | [2H]C1(C2CC3CC(C2)C(C1(C3([2H])[2H])N)([2H])[2H])[2H] | InChI=1S/C10H17N/c11-10-4-7-1-8(5-10)3-9(2-7)6-10/h7-9H,1-6,11H2/i4D2,5D2,6D2 |

Apparatus

The liquid chromatography-tandem triple quadrupole mass spectrometer 1260-6460 (Agilent, USA), multi-tube rapid mixer QB-600 (Kylin-Bell, China), Neofuge 15R high-speed refrigerated centrifuge (Shanghai Lishen Scientific Instruments Co., Ltd., China), and ultrapure water system (Millipore, USA) were used. For gradient elution, an Agilent Zorbax SB-C18 (2.1 × 100 mm, 3.5 μm), was applied using mobile phase A and B. The flow rate was set to 0.3 mL/min. The settings of the mass spectrometer were as follows: Ionization mode, Electrospray ionization (positive ion mode); operational mode, Multiple reaction monitoring (MRM); Ion source temperature, 400°C. The instrumental conditions and mobile phases parameters are listed in Supplementary Table 2 and 3.

**Supplementary Table 2:** Instrumental conditions: Precursor ion, product ions, collision energies and fragmentor of Amantadine and amantadine-d6 each. * Is for quantitative ions.

|  | **Precursor ion [m/z]** | **Product ion [m/z]** | **Collision energy**  **[eV]** | **Fragmentor**  **[eV]** |
| --- | --- | --- | --- | --- |
| Amantadine | 152.1 | 135.1* 79.2 | 16 34 | 70 |
| amantadine-d6 | 158.0 | 141.1* | 16 | 105 |

**Supplementary Table 3:** mobile phases parameters of gradient elution of amantadine.

| Time/min | Water (0.1% formic acid)/% | Acetonitrile/% |
| --- | --- | --- |
| 0.00 | 90 | 10 |
| 2.50 | 65 | 35 |
| 3.00 | 65 | 35 |
| 3.10 | 90 | 10 |
| 6.00 | 90 | 10 |

Calibrators used for the standard addition approach

A standard stock solution of amantadine was precisely prepared in methanol at a concentration of 1 mg/mL. This stock solution was subsequently diluted in a gradient fashion to prepare standard working solutions at concentrations of 100 μg/mL, 10 μg/mL, and lower as required. The internal standard, amantadine-D6, was similarly prepared in methanol at a concentration of 100 μg/mL and stored at -20°C to ensure stability.

Method validation

Six blank (no analyte, no internal standard) rats’ matrix (including heart blood, heart, liver, spleen, lung, kidney, brain, lower limb muscles, and testis) are spiked with the AMD standard solution at 20% of the lower limit of quantitation (LLOQ) concentration. These samples are processed according to the sample preparation method and analyzed by LC-MS/MS to evaluate specificity.

AMD standard solutions were spiked into 50 μL of blank rat blood to prepare calibration samples with concentrations of 1–1,000 ng/mL. Similarly, AMD standard solutions were added to 50 mg of blank rat tissues (heart, liver, spleen, lung, kidney, brain, lower limb muscles, and testis) to prepare calibration samples with concentrations of 0.1–100 ng/mg. After sample preparation, all samples were analyzed using LC-MS/MS. Calibration curves were constructed by performing linear regression, with the target analyte concentration as the x-axis and the peak area ratio of the analyte to its internal standard as the y-axis.

The limit of detection (LOD) is defined as the lowest concentration of the analyte in the spiked matrix that produces a signal-to-noise ratio (SNR) of ≥3. The lower limit of quantitation (LLOQ) is defined as the lowest concentration in the spiked matrix with an SNR of ≥10. These parameters evaluate the sensitivity of the analytical method.

AMD standard solutions were spiked into blank rat blood to prepare three concentration levels: L (10 ng/mL), M (250 ng/mL), and H (1,000 ng/mL). Similarly, AMD was spiked into blank matrixes (including heart, liver, spleen, lung, kidney, brain, lower limb muscles, and testis) to prepare three concentration levels: L (1 ng/mg), M (25 ng/mg), and H (100 ng/mg). These samples are processed according to the sample preparation method. For each concentration level, six parallel replicates were prepared. Intra-day accuracy and precision were assessed by performing three independent measurements at different times (morning, noon, and evening) on the same day. Inter-day accuracy and precision were evaluated by performing two independent measurements at noon on each of the following two days. Accuracy and precision were determined using the calibration curve. Accuracy was quantified as the relative recovery (Rel. R%), calculated as the percentage of the measured value relative to the theoretical value. Precision was expressed as the coefficient of variation (CV%).

The recovery of AMD from rat matrixes (including heart blood, heart, liver, spleen, lung, kidney, brain, lower limb muscles, and testis) during the extraction process was determined by comparing the analyte concentrations in pre-extraction spiked samples with those in post-extraction spiked samples at three concentration levels (L, M, and H; n = 6). To evaluate the matrix effect, blank matrixes samples were extracted and spiked with AMD standards (n = 6). The matrix effect was assessed by comparing the peak area of AMD in matrix-spiked samples (A) with that in water-substituted samples at the same concentrations (B). The matrix effect was expressed as the percentage ratio of A to B. A value of 100% indicates no matrix effect, values > 100% indicate ionization enhancement, and values < 100% indicate ionization suppression.

Sample preparation was consistent with that used for accuracy and precision assessments, with three sets of samples analyzed. The first set was processed and analyzed immediately after preparation. The second set was subjected to three freeze-thaw cycles (-20°C to room temperature) before processing and analysis. The third set was analyzed 24 hours after being processed and stored in the autosampler. Freeze-thaw stability and autosampler stability were evaluated based on deviation, calculated as: Deviation (%) = (True concentration - Measured concentration) / True concentration × 100.

AMD standards were spiked into blank rat blood to prepare samples at concentrations of 10 μg/mL and 100 μg/mL, with six parallel replicates per group. Similarly, AMD standards were spiked into other blank matrixes (heart, liver, spleen, lung, kidney, brain, lower limb muscles, and testis) to prepare samples at concentrations of 1 μg/mg and 10 μg/mg, with six parallel replicates per group. The spiked blood or liver samples were then diluted 10-fold and 100-fold with blank rat blood or liver, respectively, and processed according to the sample preparation method for analysis. The dilution effect was assessed in terms of accuracy and precision.

**Supplementary Table 4:** Analytical method validation results for AMD in rat blood and tissues assay.

|  | Liner | | LOD  (ng/mL or ng/mg) | LLOQ  (ng/mL or ng/mg) | Conc. | Accuracy  (RE %) | | Precision  (CV %) | | Recovery  (%) | Matrix effect (%) | Stability  (%) | | Dilution Effects (%) | |
| --- | --- | --- | --- | --- | --- | --- | --- | --- | --- | --- | --- | --- | --- | --- | --- |
|  | equation | *r* |  |  |  | Intra-day | Inter-day | Intra-day | Inter-day |  |  | freeze and thaw | on-instrument | Accuracy  (RE %) | Precision  (CV %) |
| Blood | y = 0.091631*x + 1.061372 | 0.9957 | 5.0 | 10 | L | 89.2 ± 4.4 | 90.2 ± 5.8 | 7.3 ± 1.1 | 10.3 ± 2.2 | 90.2 ± 2.4 | 82.1 ± 5.2 | 7.2 ± 2.2 | 6.5 ± 0.5 | 104.4±9.2 | 6.7±2.5 |
|  |  |  |  |  | M | 88.5 ± 2.1 | 89.5 ± 6.4 | 6.1 ± 0.6 | 5.3 ± 0.6 | 93.1±2.1 | 77.3±4.7 | 3.8 ± 0.8 | 2.8 ± 0.6 |  |  |
|  |  |  |  |  | H | 91.3 ± 6.2 | 89.1 ± 5.6 | 3.7 ± 0.7 | 2.7 ± 0.8 | 94.4±7.3 | 87.1±5.1 | 4.5 ± 2.4 | 5.1 ± 2.4 |  |  |
| Heart | y = 0.241734*x + 0.901133 | 0.9951 | 0.5 | 1.0 | L | 99.2 ± 2.5 | 88.2 ± 2.2 | 5.3 ± 0.4 | 10.3 ± 2.5 | 88.2±5.8 | 88.2±5.8 | 7.2 ± 3.6 | 10.5 ± 2.5 | 102.5±9.8 | 11.9±3.5 |
|  |  |  |  |  | M | 97.5 ±4.7 | 94.5 ± 6.2 | 9.6 ± 2.6 | 2.3 ± 0.6 | 86.1±3.2 | 84.7±3.1 | 9.8 ± 3.8 | 6.8 ± 2.6 |  |  |
|  |  |  |  |  | H | 90.2 ± 4.6 | 93.3 ± 2.5 | 2.6 ± 1.4 | 6.7 ± 0.2 | 87.2±2.7 | 83.2±6.2 | 2.5 ± 1.4 | 7.1 ± 3.4 |  |  |
| liver | y = 0.175184*x + 4.053114 | 0.9972 | 0.5 | 1.0 | L | 103.2 ± 6.4 | 90.2 ± 5.8 | 5.3 ± 1.1 | 9.3 ± 2.5 | 87.7±5.2 | 79.6±6.1 | 11.2 ± 3.5 | 5.5 ± 2.5 | 97.5±6.2 | 7.2±3.7 |
|  |  |  |  |  | M | 87.5 ±1.7 | 92.5 ± 6.4 | 6.6 ± 3.6 | 5.3 ± 0.6 | 83.5±5.3 | 80.3±5.4 | 8.8 ± 3.7 | 6.9 ± 2.5 |  |  |
|  |  |  |  |  | H | 91.3 ± 6.2 | 99.1 ± 5.6 | 3.6 ± 1.4 | 6.7 ± 0.2 | 88.1±6.9 | 79.9±2.6 | 6.5 ± 3.4 | 7.2 ± 3.4 |  |  |
| Spleen | y = 0.082766*x + 2.001847 | 0.9991 | 0.25 | 0.5 | L | 104.2 ± 5.5 | 96.2 ± 4.2 | 8.3 ± 3.4 | 9.2 ± 3.5 | 82..4±6.9 | 80.1±7.1 | 7.2 ± 3.5 | 6.5 ± 2.5 | 104.4±9.2 | 6.7±2.3 |
|  |  |  |  |  | M | 109.5 ±2.7 | 97.5 ± 4.2 | 8.6 ±2.6 | 5.3 ± 2.6 | 84.3±3.1 | 87.9±6.3 | 4.5 ± 1.7 | 2.9 ± 0.5 |  |  |
|  |  |  |  |  | H | 92.2 ± 4.7 | 92.3 ± 5.5 | 5.6 ± 2.7 | 7.7 ± 1.2 | 85.2±4.1 | 85.2±4.1 | 7.5 ± 2.4 | 5.2 ± 3.4 |  |  |
| Lung | y = 0.095739*x + 1.863261 | 0.9983 | 0.25 | 0.5 | L | 110.2 ± 2.4 | 89.2 ± 5.0 | 7.3 ± 1.1 | 10.3 ± 2.5 | 86.1±3.6 | 87.1±5.1 | 11.2 ± 3.5 | 5.5 ± 2.5 | 99.3±4.8 | 10.3±4.5 |
|  |  |  |  |  | M | 92.5 ±1.3 | 94.5 ± 6.2 | 6.6 ± 3.6 | 2.3 ± 0.6 | 87.2±5.4 | 86.4±4.7 | 4.5 ± 1.7 | 7.9 ± 2.3 |  |  |
|  |  |  |  |  | H | 91.3 ± 6.6 | 103.1 ± 2.6 | 2.6 ± 1.4 | 6.7 ± 0.2 | 85.2±6.2 | 85.1±7.1 | 6.5 ± 2.4 | 8.2 ± 2.4 |  |  |

**Supplementary Table 4:** Analytical method validation results for AMD in rat blood and tissues assay (Continued)

|  | Liner | | LOD  (ng/mL or ng/mg) | LLOQ  (ng/mL or ng/mg) | Conc. | Accuracy  (RE %) | | Precision  (CV %) | | Recovery  (%) | Matrix effect (%) | Stability  (%) | | Dilution Effects (%) | |
| --- | --- | --- | --- | --- | --- | --- | --- | --- | --- | --- | --- | --- | --- | --- | --- |
|  | equation | *r* |  |  |  | Intra-day | Inter-day | Intra-day | Inter-day |  |  | freeze and thaw | on-instrument | Accuracy  (RE %) | Precision  (CV %) |
| kidney | y = 0.231173*x + 0.094725 | 0.9963 | 5.0 | 10 | L | 89.2 ± 4.4 | 90.2 ± 5.8 | 11.2 ± 2.2 | 6.5 ± 0.5 | 92.3 ± 6.2 | 99.1 ± 5.6 | 7.2 ± 3.5 | 6.5 ± 0.5 | 105.0±4.2 | 5.7±1.3 |
|  |  |  |  |  | M | 88.5 ± 2.1 | 89.5 ± 6.4 | 3.8 ± 0.8 | 10.8 ± 0.6 | 104.2 ± 5.5 | 98.2 ± 4.2 | 6.8 ± 2.8 | 2.8 ± 0.6 |  |  |
|  |  |  |  |  | H | 91.3 ± 6.2 | 89.1 ± 5.6 | 6.5 ± 2.4 | 9.1 ± 2.4 | 105.5 ±2.7 | 95.5 ± 4.2 | 5.5 ± 1.4 | 5.1 ± 2.4 |  |  |
| brain | y = 0.082641*x + 0.061372 | 0.9958 | 0.5 | 1.0 | L | 89.2 ± 4.4 | 90.2 ± 5.8 | 6.5 ± 3.4 | 6.2 ± 3.4 | 89.3 ± 4.4 | 90.2 ± 5.8 | 4.1 ± 2.4 | 2.7 ± 0.3 | 99.0±7.4 | 6.7±2.2 |
|  |  |  |  |  | M | 88.5 ± 2.1 | 89.5 ± 6.4 | 7.3 ± 3.5 | 6.5 ± 2.5 | 88.7 ± 2.1 | 89.5 ± 5.4 | 9.8 ± 3.8 | 11.5 ± 3.5 |  |  |
|  |  |  |  |  | H | 91.3 ± 6.2 | 89.1 ± 5.6 | 8.5 ± 1.7 | 2.9 ± 2.5 | 91.3 ± 6.2 | 89.1 ± 4.6 | 3.9 ± 0.8 | 7.5 ± 1.4 |  |  |
| lower limb muscles | y = 0.193581*x + 0.074591 | 0.9990 | 0.25 | 0.5 | L | 89.2 ± 4.4 | 90.2 ± 5.8 | 3.5 ± 1.7 | 2.9 ± 0.5 | 88.3±3.1 | 87.9±6.3 | 6.5 ± 2.6 | 7.2 ± 3.4 | 95.3±6.2 | 4.7±2.3 |
|  |  |  |  |  | M | 88.5 ± 2.1 | 89.5 ± 6.4 | 7.5 ± 2.4 | 6.2 ± 3.4 | 85.2±3.7 | 89.2±4.1 | 7.2 ± 2.4 | 4.5 ± 1.7 |  |  |
|  |  |  |  |  | H | 91.3 ± 6.2 | 89.1 ± 5.6 | 11.0 ± 3.5 | 7.5 ± 2.5 | 94.4±7.3 | 87.1±5.1 | 4.5 ± 2.0 | 6.5 ± 3.4 |  |  |
| testis | y = 0.076321*x + 1.131302 | 0.9973 | 0.5 | 1.0 | L | 89.2 ± 4.4 | 90.2 ± 5.8 | 8.8 ± 3.3 | 6.9 ± 2.5 | 87.2±4.9 | 88.2±7.3 | 4.6 ± 2.7 | 3.7 ± 0.8 | 101.4±3.2 | 9.2±4.3 |
|  |  |  |  |  | M | 88.5 ± 2.1 | 89.5 ± 6.4 | 5.5 ± 3.4 | 6.2 ± 3.4 | 89.3 ± 4.1 | 91.2 ± 5.8 | 5.3 ± 2.4 | 3.8 ± 1.6 |  |  |
|  |  |  |  |  | H | 91.3 ± 6.2 | 89.1 ± 5.6 | 7.2 ± 3.5 | 12.5 ± 2.5 | 88.5 ± 2.2 | 99.5 ± 6.4 | 6.7 ±1.5 | 8.8 ± 3.7 |  |  |

**Supplementary Table 5:** The concentrations of AMD in each tissue at different time points in the exposed rats (μg/mL/ng/mg，n=6).

| Specimen | 0.5h | 1h | 2h | 4h | 8h | 12h | 24h | 48h | 72h | 96h |
| --- | --- | --- | --- | --- | --- | --- | --- | --- | --- | --- |
| cardiac blood | 8. 13±1.85 | 12.97±3.66* | 9.85±3.01 | 10.56±3.84 | 7.86±2.44 | 4.52±0.74 | 1.92±0.24 | 0.73±0.15 | 0.21±0.05 | 0.04±0.02 |
| Heart | 92.22±34.58 | 134.54±55.33 | 217.34±51.30* | 182.44±44.72 | 180.27±80.12 | 131.08±58.40 | 101.31±29.82 | 80.65±15.52 | 43.93±12.22 | 16.62±9.30 |
| Liver | 138. 12±30.41 | 166.37±63.64 | 357.75±95.80* | 287. 13±61.32 | 248.26±63.63 | 228.54±80.33 | 178.71±41.13 | 135.30±45.71 | 105.62±32.81 | 32.64±7.37 |
| Spleen | 81.99±25.73 | 134.74±45.71 | 217.45±47.71* | 150.51±33.90 | 132.55±34.61 | 117.55±19.90 | 123.66±30.61 | 89.92±24.42 | 60.88±12.47 | 13.34±4.21 |
| Lung | 96.07±28.80 | 116.21±25.70 | 192.20±45.67 | 197.34±57.21* | 160.52±42.73 | 143.35±29.91 | 124.47±38.24 | 94.03±22.53 | 55.71±14.25 | 12.44±3.14 |
| Kidney | 70.22±16.71 | 126.51±33.66 | 281.26±64.13 | 353.48±87.38* | 264.55±99.80 | 223. 1±71.71 | 161.27±27.42 | 129.21±47.54 | 85.99±19.31 | 22.37±5.94 |
| Brain | 34.42±10.31 | 69. 12±17.14 | 93.27±29.51* | 87.88±18.11 | 86.48±18.11 | 73.68±14.54 | 59.93±15.65 | 43.91±11.42 | 18.64±5.28 | 6.35±2.19 |
| Muscles | 19.95±5.05 | 42. 16±9.42 | 67.37±11.56 | 79.95±8.55* | 63.75±10.25 | 53.36±8.53 | 33. 11±7.07 | 13.32±4.21 | 1.01±0.71 | # |
| Testis | 19.01±4.18 | 34.74±7.49 | 45.84±8.71 | 47.02±10.80* | 32.55±7.92 | 22.51±7.37 | 15.63±3.17 | 9.49±3.27 | 0.85±0.72 | # |

*: AMD concentration at this time point denotes the peak value (p<0.05)；#: The AMD concentration at the time point is lower than the LLOQ.

| Tissues | Temperature | C_6h_/C_0h_ | | | C_12h_/C_0h_ | | | C_24h_/C_0h_ | | | C_48h_/C_0h_ | | | C_72h_/C_0h_ | | | C_96h_/C_0h_ | | |
| --- | --- | --- | --- | --- | --- | --- | --- | --- | --- | --- | --- | --- | --- | --- | --- | --- | --- | --- | --- |
|  |  | L | M | H | L | M | H | L | M | H | L | M | H | L | M | H | L | M | H |
| Peripheral  blood | 4℃ | 1.27 | 0.82 | 0.79 | 1.50 | 0.83 | 0.96 | 1.63 | 1.77 | 1.21 | 1.71 | 2.81 | 1.91 | NA | NA | NA | NA | NA | NA |
|  | 20℃ | 1.49 | 0.83 | 0.64 | 1.73 | 0.63 | 0.68 | 2.11 | 0.31 | 0.94 | 2.45 | 4.70 | 1.70 | NA | NA | NA | NA | NA | NA |
| Cardiac  blood | 4℃ | 1.06 | 1.02 | 2.27 | 1.26 | 1.01 | 3.01 | 1.44 | 1.13 | 2.30 | 1.19 | 1.43 | 2.55 | 1.44 | 1.60 | 3.01 | NA | NA | NA |
|  | 20℃ | 0.76 | 0.72 | 0.67 | 0.87 | 0.75 | 0.61 | 1.06 | 0.28 | 0.73 | 1.06 | 1.17 | 1.10 | 1.55 | 1.55 | 2.26 | NA | NA | NA |
| Heart | 4℃ | 1.20 | 0.59 | 1.45 | 0.87 | 0.45 | 1.52 | 1.36 | 0.59 | 1.02 | 0.74 | 0.97 | 1.02 | 1.29 | 0.69 | 1.32 | 1.59 | 0.86 | 1.16 |
|  | 20℃ | 0.70 | 0.59 | 0.64 | 0.95 | 0.52 | 0.61 | 1.06 | 0.17 | 1.09 | 0.98 | 1.21 | 0.69 | 1.26 | 1.07 | 0.93 | 1.38 | 1.44 | 1.19 |
| Liver | 4℃ | 0.97 | 0.75 | 2.69 | 0.83 | 0.75 | 2.46 | 1.03 | 1.91 | 2.19 | 0.62 | 2.31 | 2.92 | 1.41 | 1.63 | 3.57 | 2.01 | 2.00 | 4.29 |
|  | 20℃ | 0.52 | 0.75 | 1.05 | 0.56 | 0.88 | 0.89 | 0.65 | 0.72 | 1.52 | 0.62 | 3.75 | 2.54 | 1.27 | 4.06 | 2.73 | 2.32 | 4.44 | 3.69 |
| Spleen | 4℃ | 1.42 | 0.81 | 1.92 | 1.07 | 0.86 | 3.72 | 1.39 | 0.86 | 2.58 | 0.98 | 1.22 | 2.42 | 1.67 | 1.57 | 4.81 | 2.21 | 1.86 | 6.14 |
|  | 20℃ | 0.84 | 1.19 | 3.19 | 1.13 | 0.81 | 2.97 | 0.88 | 0.49 | 3.33 | 1.35 | 4.89 | 4.67 | 3.45 | 3.92 | 6.44 | 5.12 | 4.56 | 6.06 |
| Lung | 4℃ | 1.21 | 0.69 | 1.02 | 0.72 | 0.69 | 0.94 | 0.87 | 0.69 | 0.55 | 0.66 | 1.14 | 0.71 | 0.75 | 0.86 | 0.71 | 1.03 | 1.07 | 0.55 |
|  | 20℃ | 0.63 | 0.69 | 0.35 | 0.47 | 0.62 | 0.32 | 0.39 | 0.28 | 0.39 | 0.89 | 1.24 | 0.18 | 0.37 | 1.31 | 0.33 | 0.64 | 1.41 | 0.55 |
| Kidney | 4℃ | 1.08 | 0.97 | 1.96 | 0.96 | 0.68 | 1.98 | 1.30 | 1.00 | 1.86 | 0.87 | 1.25 | 1.96 | 1.21 | 1.37 | 2.25 | 1.55 | 1.55 | 2.08 |
|  | 20℃ | 0.72 | 0.95 | 0.64 | 1.04 | 0.81 | 0.64 | 0.57 | 0.53 | 0.90 | 0.70 | 3.83 | 2.47 | 1.07 | 4.63 | 2.49 | 1.94 | 3.95 | 2.33 |
| Brain | 4℃ | 1.22 | 1.11 | 0.90 | 0.82 | 1.56 | 0.86 | 1.39 | 1.22 | 0.52 | 0.75 | 2.33 | 0.81 | 1.33 | 1.44 | 0.81 | 1.74 | 1.56 | 0.95 |
|  | 20℃ | 0.96 | 1.22 | 0.48 | 1.02 | 1.06 | 0.52 | 0.74 | 0.78 | 0.43 | 0.95 | 1.67 | 0.33 | 1.01 | 2.44 | 0.33 | 1.45 | 2.56 | 0.76 |
| Muscle | 4℃ | 1.46 | 1.00 | 0.98 | 1.12 | 0.92 | 0.82 | 1.79 | 1.00 | 0.94 | 1.13 | 1.83 | 0.59 | 1.74 | 1.08 | 1.06 | 2.27 | 1.33 | 1.29 |
|  | 20℃ | 1.06 | 1.08 | 0.88 | 1.43 | 0.83 | 0.82 | 1.13 | 0.42 | 0.76 | 1.42 | 2.08 | 0.59 | 1.52 | 1.33 | 0.71 | 2.34 | 2.08 | 1.35 |
| Testis | 4℃ | 1.12 | 1.00 | 1.83 | 0.97 | 1.40 | 1.46 | 1.37 | 1.30 | 1.28 | 1.27 | 1.80 | 1.33 | 1.70 | 1.40 | 1.39 | 2.01 | 1.60 | 1.83 |
|  | 20℃ | 0.97 | 1.21 | 2.00 | 0.93 | 0.95 | 1.93 | 1.30 | 0.58 | 1.55 | 1.34 | 2.43 | 1.04 | 2.18 | 2.66 | 2.01 | 2.34 | 2.78 | 2.17 |

**Supplementary Table 6:** Ratio of AMD concentration in each tissue of rats at different time points to the concentration at the time of death
